# hMSCs as an alternative therapeutic option for asthma with neutrophil mediated inflammation

**DOI:** 10.1038/s12276-018-0072-7

**Published:** 2018-06-07

**Authors:** Fernanda F. Cruz, Patricia R. M. Rocco, Daniel J. Weiss

**Affiliations:** 1 Laboratory of Pulmonary Investigation, Carlos Chagas Filho Biophysics Institute, Federal University of Rio de Janeiro, Rio de Janeiro, Brazil; 2 Vermont Lung Center, College of Medicine, University of Vermont, Burlington, VT USA

We read with great interest the recently published manuscript, “hMSCs suppress neutrophil-dominant airway inflammation in a murine model of asthma” by Hong and colleagues (10.1038/emm.2016.135). This study aimed to test human mesenchymal stem cells (hMSCs) as an alternative therapeutic option for asthma with neutrophil dominant inflammation. Even though the authors claim that hMSCs have been used for the first time in this model of Poly (I-C)-induced neutrophilic airway inflammation, there have been a number of publications investigating the effect of systemically administered MSCs, including hMSCs, in other mouse models of Th-17 neutrophilic allergic airways inflammation^1–4^. Our group has published 2 recent papers in this area (Cruz et al., 2015a, b), and neither of them are referenced or discussed^2,3^. As this is a rapidly evolving field, with important functional, morphological and immunological changes, it is important to provide more current references and discussion.

With this letter, we further request that the authors clarify and expand discussion on the following issues: (1) the use of hMSCs in immunocompetent mouse models; (2) the absence of measurements of lung mechanics and their correlation with lung histology and inflammation; (3) the need of quantitative histologic injury scores to validate the qualitative histologic images in Figure 1b. It is confusing why there is more apparent histologic inflammation in mice that received high dose compared to low dose of hMSCs; (4) cells fluorescing in green are described as MSCs in Figure 2. Nevertheless, the cells administered were heterogeneous mixtures and the antibody used for detection was human B-microglobulin. As such, there is no evidence that the cells are MSCs. Additionally, hMSCs require characterization for their stemness and differentiation capabilities; (5) it is unclear why interleukin (IL)-17 was not detected in BALF. Neutrophilic severe asthma has a strong Th-17 component. Even though IL-5 is an eosinophilic chemoattractant, in this model, IL-5 as well as IL-4 and IL-13 seem to be irrelevant, (6) Moreover, the relevance of IL-4 and IL-13 in the serum monocytes in severe neutrophilic asthma is also unclear, (7) so far, there has been no convincing evidence that MSCs actually migrate, and 8) Indoleamine 2,3-dioxygenase (IDO) is not a secreted molecule.

Although we agree with the authors the importance of evaluating the role of hMSCs in neutrophilic asthma; all these parameters described above should be better discussed. Further studies should be conducted to ascertain whether hMSCs have similar beneficial effects in other models of neutrophilic asthma.

## The authors reply:

You Sook Cho

Division of Allergy and Clinical Immunology, Department of Internal Medicine, Asan Medical Center, University of Ulsan College of Medicine, 88, Olympic-ro 43-gil, Songpa-gu, Seoul 138-736, Korea.

We greatly thank Cruz et al. for their interest in our paper. We agree with their view that we should have provided more current references and discussion in our article, and also regret that we have omitted the valuable references they have mentioned. We are glad we have added evidences that MSCs could attenuate airway inflammation in asthma, similar to what they have studied for several years.

The umbilical cord-derived hMSCs were provided by MEDIPOST Co. (Co-author Wonil Oh). The hMSCs from MEDIPOST have been well studied in immune-competent mice. We have used human MSCs in immune-competent mice as most studies have done successfully in showing the immune-modulatory effect of hMSCs. The MSCs were well characterized regarding their purity, stemness, and differentiation capabilities, as shown elsewhere in many studies using MEDIPOST MSCs.

We concur with Cruz et al. that measuring AHR and unbiased scoring of inflammation in the histology could have made the description of the asthmatic phenotype more solid. In fact, we have measured AHR in our other published studies regarding asthma severity and phenotype characterizations. However, in this study, we have focused on the effect of hMSC on the inflammation itself and hence have not measured the AHR. The decrease in inflammation on histology of MSC-treated mice was very obvious, hence we did not show the inflammatory score. The attenuation of inflammation was significant even on treatment with low-dose hMSC, and the difference between low- and high-dose hMSC treatment was not very large. Thus, histology sections of low- and high-dose hMSC-treated mice were similar, and the presented sections show similar inflammation levels, which were both much lower than that for the non-treated mice. In reality, the amount of inflammation in mice lung is not consistent throughout the lung, but vary at different locations. Thus, some parts of the high-dose hMSC-treated mice lung may look more severe than those of low-dose hMSC-treated mice lung, but overall the the inflammation was slightly less in high-dose hMSC-treated mice. Yet, the main result that hMSC decreased neutrophilic inflammation still remains unaffected despite the absence of the additional data.

We agree that there has been no concrete evidence that MSCs actually migrate into lung tissue. We speculate that trapped lung MSC could modulate peripheral lung inflammation by local production of anti-inflammatory cytokines or by production of extracellular vesicles. However, our data show that human β-microglobulin-stained cells were mostly stained around the highly inflammed peribronchial/perivascular area. Considering the high purity of MSCs from MEDIPOST Co., we hypothesize that there could be migration of hMSCs into inflamed tissue, although additional studies are required to support this evidence.

The studies by Weiss et al. have well shown that mesenchymal stromal cells suppressed mixed TH2/TH17 inflammation in aspergillus hyphal extract (AHE)-induced allergic airway inflammation. However, we have used a different asthma model mimicking viral infection by using poly-IC. Poly-IC itself stimulates lung epithelial cells, induces IL-8, stimulates allergen-specific TH1 cells, and induces severe neutrophilic inflammation. It is unclear why IL-17 was not detected in the BALF. We speculate that IL-17 production was lower and CXCL15 response was higher in the airways of our mice model compared to the AHE-induced allergic airway inflammation. However, IL-17 was highly secreted in stimulated cells from lung-draining LNs of asthmatic mice showing the presence of TH17 cells. Treatment with high-dose hMSC decreased the IL-17 production in stimulated LN cells. Moreover, we also showed that production of IL-8 in BEAS2-B cells in response to poly-IC-stimulation was reduced after co-culture with hMSCs. These data suggested that hMSC could attenuate neutrophilic inflammation by suppressing both IL-17 and CXCL15 in our poly-IC-stimulated asthma model.

Because the purpose of our study was to observe the effect of hMSC in a highly neutrophil-dominant mice model, we agree that TH2 cytokines such as IL-4 and IL-13 are less relevant. However, the enrolled severe asthma patients showed mixed granulocytic inflammation in their sputum and consequently the stimulated PBMCs from these severe asthmatics produced both IL-4 and IL-13 as well as IFN-. Human asthmatic studies have shown that IFN- is highly produced in virus-induced asthma exacerbation and induces severe neutrophilic inflammation.
